# Ectomycorrhizal Fungal Communities and Their Functional Traits Mediate Plant–Soil Interactions in Trace Element Contaminated Soils

**DOI:** 10.3389/fpls.2018.01682

**Published:** 2018-11-20

**Authors:** Marta Gil-Martínez, Álvaro López-García, María T. Domínguez, Carmen M. Navarro-Fernández, Rasmus Kjøller, Mark Tibbett, Teodoro Marañón

**Affiliations:** ^1^Department for Protection of the Soil, Plant and Water System, Institute of Natural Resources and Agrobiology of Seville, Spanish National Research Council, Seville, Spain; ^2^Department of Biology, University of Copenhagen, Copenhagen, Denmark; ^3^Área de Edafología y Química Agricola, Departamento de Cristalografía, Mineralogía y Química Agrícola, Universidad de Sevilla, Seville, Spain; ^4^Centre for Agri-Environmental Research and Soil Research Centre, School of Agriculture, Policy and Development, University of Reading, Reading, United Kingdom

**Keywords:** ecosystem processes, heavy metal, microbiome, phytoremediation, *Quercus ilex* subsp. *ballota* (holm oak), root economics spectrum, symbiosis, trace element transfer

## Abstract

There is an increasing consensus that microbial communities have an important role in mediating ecosystem processes. Trait-based ecology predicts that the impact of the microbial communities on ecosystem functions will be mediated by the expression of their traits at community level. The link between the response of microbial community traits to environmental conditions and its effect on plant functioning is a gap in most current microbial ecology studies. In this study, we analyzed functional traits of ectomycorrhizal fungal species in order to understand the importance of their community assembly for the soil–plant relationships in holm oak trees (*Quercus ilex* subsp. *ballota*) growing in a gradient of exposure to anthropogenic trace element (TE) contamination after a metalliferous tailings spill. Particularly, we addressed how the ectomycorrhizal composition and morphological traits at community level mediate plant response to TE contamination and its capacity for phytoremediation. Ectomycorrhizal fungal taxonomy and functional diversity explained a high proportion of variance of tree functional traits, both in roots and leaves. Trees where ectomycorrhizal fungal communities were dominated by the abundant taxa *Hebeloma cavipes* and *Thelephora terrestris* showed a conservative root economics spectrum, while trees colonized by rare taxa presented a resource acquisition strategy. Conservative roots presented ectomycorrhizal functional traits characterized by high rhizomorphs formation and low melanization which may be driven by resource limitation. Soil-to-root transfer of TEs was explained substantially by the ectomycorrhizal fungal species composition, with the highest transfer found in trees whose roots were colonized by *Hebeloma cavipes*. Leaf phosphorus was related to ectomycorrhizal species composition, specifically higher leaf phosphorus was related to the root colonization by *Thelephora terrestris*. These findings support that ectomycorrhizal fungal community composition and their functional traits mediate plant performance in metal-contaminated soils, and have a high influence on plant capacity for phytoremediation of contaminants. The study also corroborates the overall effects of ectomycorrhizal fungi on ecosystem functioning through their mediation over the plant economics spectrum.

## Introduction

There is an increasing consensus that microbial communities have an important role in mediating ecosystem processes. In recent years, and thanks to the development of molecular approaches, several studies have focused on the interaction between plants and soil microbial communities to reveal the potential of microbes to drive vegetation diversity and dynamics (Bever, 2003; Wardle et al., 2004; van der Heijden et al., 2015; Erktan et al., 2018; Rutten and Gómez-Aparicio, 2018). As vegetation determines how ecosystems function to a large extent, plant microbiomes indirectly affect the provision of multiple ecosystem services (Friesen et al., 2011; Van der Putten et al., 2013). In addition, some studies have highlighted the existence of feedback processes between plants and soil organisms (Bever et al., 2010; Brinkman et al., 2010), suggesting not only the potential of microbes to modify plant communities but also the role of plant communities and their traits at structuring microbial community compositions (de Vries et al., 2012; Aponte et al., 2013; Bauman et al., 2016; López-García et al., 2017).

Although the effect of plant hosts on their microbiomes has often been studied from a taxonomic point of view (Aponte et al., 2010; de Vries et al., 2012; Kurm et al., 2018), little is known about how soil microbial functional traits are affecting the functioning of plant species. It is debatable whether the features of microbes associated to individual plants (species composition and trait distribution) can be actually defined as plant traits, as they are not heritable features, according to the definition of Garnier et al. (2016). Often, microbial traits in the root microbiome are referred as “biotic root traits” (Bardgett et al., 2014). Recently, some authors have considered the use of traits in the root microbiome as an extension of the plant species phenotype for explaining functional changes in plant communities along environmental gradients and it has been included in a multidimensional root trait framework (Navarro-Fernández et al., 2016; Weemstra et al., 2016).

The influence of the plant microbiome from a trait-based perspective usually requires assessment of individual species in communities (Díaz et al., 2007), and this has been proven to be very challenging when working with microbes (see Crowther et al., 2014). According to the current thinking on ecological assembly, recording traits at individual species level will allow to differentiate between response and effect traits (Zirbel et al., 2017). The links between the response of microbial community-level traits to environmental conditions and the effects of these microbial traits changes on plant functioning is an important knowledge gap to be filled in current microbial ecology studies, although the existence of these links have been predicted previously (see Koide et al., 2014).

Mycorrhizal fungi are recognized for their importance for plant foraging of soil resources (Tibbett and Sanders, 2002; van der Heijden et al., 2015; Köhler et al., 2018), particularly in plant species with relatively thick absorptive roots (Eissenstat et al., 2015; Liu et al., 2015). Coevolution of plant and fungal partners has been recently suggested by Chen et al. (2018), based on their description of a root-fungal functional complementarity in nutrient foraging. However, how mycorrhizal and plant traits are interrelated, for example aligned into the common root economics spectrum framework, and how mycorrhizal traits mediates soil–plant relationships are still open questions that need to be addressed (Weemstra et al., 2016).

This mycorrhiza–root association improves plant health by enhancing resistance to diverse stresses like drought, salinity, heavy metals and pathogens, among others (van der Heijden et al., 2015). Therefore, mycorrhizal mediation on plant performance might be especially important in highly stressful environments, such as trace element (TE) contaminated soils. In these soils, mycorrhizal fungi enhance plant nutrition, stress tolerance and soil structure and, consequently, promote the recovery of the functions in the degraded soil (van der Heijden and Scheublin, 2007; Firmin et al., 2015). Association with mycorrhizal fungi can also play an important role in the transfer of TEs through the soil–root continuum, an issue of special relevance for the management of TE contaminated sites. For instance, the phytostabilization approach is a phytoremediation technology that combines the use of soil amendments and plants to immobilize pollutants into the soil, thus reducing the risks of transfer of these pollutants through the aboveground food web (Mendez and Maier, 2008). A prerequisite to apply this approach to large contaminated areas is that the plants used to remediate the soil can retain TEs at the rhizosphere level, and do not accumulate them into their aboveground biomass (Bolan et al., 2011; Madejón et al., 2018a). In relation to this, ectomycorrhizal (ECM) fungi may provide protection against metal toxicity through avoidance (i.e., extracellular precipitation, biosorption to cell walls, reduced uptake) and sequestration (i.e., intracellular chelation, compartmentation into fungi vacuoles) (Hartley et al., 1997; Jentschke and Godbold, 2000; Bellion et al., 2006). Therefore, phytoremediation of TE polluted soils can be facilitated by ECM fungi as they adapt to TE stress promoting the host growth (Wen et al., 2017).

In this study, we aimed to elucidate the role of ECM community in the plant nutritional status and the transfer of TEs through the soil-root-leaf continuum in a large-scale phytoremediation case study. Holm oaks (*Quercus ilex* subsp. *ballota*) root and leaf functional traits were analyzed in trees growing on remediated soils exhibiting a gradient of anthropogenic TE contamination. Relationships between nutrient/TE concentrations in plants and the structure on the ECM communities were evaluated. Ectomycorrhizal community composition and morphological traits along the same gradient of soil contamination were previously reported by López-García et al. (2018). Here, relationships between ECM, soil, root, and leaf variables were explored in order to understand the importance of the ECM community assembly in the soil–plant relationships in holm oak trees.

We hypothesized that (i) plant traits (i.e., morphological and chemical) of holm oak would change along TE gradient; (ii) ECM fungal communities, would partly mediate plant response to TEs, and thus a significant fraction of the plant nutrient status and transfer of TEs from soils to leaves will be explained by ECM variables (either species composition or functional traits) (iii) ECM fungal communities lead the intraspecific variation of root functional traits.

## Materials and Methods

### Study Area

The study was conducted at the Guadiamar Green Corridor (SW Spain), an area affected by a large mining accident in 1998 (the Aznalcóllar mine spill; Madejón et al., 2018b). The failure of a large tailings storage facility was one of the largest mining accidents in Europe to the date, which provoked the release of ca. 6 hm^3^ of metalliferous tailings (water and sludge) over 55 km^2^ of the Guadiamar River basin. As a result, soils were severely polluted with several TEs, mainly As, Cd, Cu, Pb, Tl, and Zn (Cabrera et al., 1999). After the accident, a large scale soil remediation program was conducted, which included the removal of the deposited sludge and the soil surface using heavy machinery, followed by the application of organic matter and calcium-rich amendments to immobilize TEs into the soil. The affected lands, mostly under agricultural production until the mining accident, were purchased by the Regional Administration, and then afforested using native tree and shrub species (Domínguez et al., 2008). Despite these remediation operations, contamination levels were highly variable across short distances in the Corridor and some patches are still highly degraded, due to acid drainage of the remnants of the sludge that lead to soil acidification and to a high solubility of toxic TEs (Domínguez et al., 2016).

The climate of the study area is typically Mediterranean, with mild rainy winters and warm dry summers. Average annual temperature is 19°C (minimum monthly mean of 9°C in January, and maximum of 27°C in July) and annual average rainfall is 484 mm. The study area harbors soils with different geology adding additional variation to the patchily distributed levels of TEs. Typical bedrock types at the North of the Corridor are slate and schist, and derived soils are naturally acidic. In the South (further than 15 km away from the mine) geological substrate tends to be dominated by calcarenite and marls originating neutral to calcareous loam soils. Potential vegetation is dominated by sclerophyllous Mediterranean forests, in particular by ECM holm oak in the alluvial terraces.

### Sampling Design

The study was conducted in April 2016, 16 years after the application of soil amendments and the plantation of the former agricultural lands with native trees and shrubs. Holm oak was the target species of the study, given that it was intensively used to afforest the alluvial terraces of the affected area. Four sites were selected along a gradient of soil pollution across the affected area. A site location map and a general description of these soils as well as their classification is provided in López-García et al. (2018). Site 1 and Site 3 were located at the North of the corridor, while Site 2 and site 4 were located at the South of the Corridor. At each site, 10 holm oak trees were randomly selected (*N* = 40 trees). All these trees were planted at the same time (Autumn 2000) and with similar seed provenance.

For each tree, roots (and their associated ECM fungi) were sampled by carefully tracing from the stems of the tree to the roots belowground in the four cardinal directions. Around 200 g of root material was collected from each direction, i.e., subsamples. Root samples were used to characterize the main root functional traits and the ECM community (see López-García et al., 2018 for ECM characterization). Soil samples (0–20 cm depth) were taken with an auger from the four directions under each tree canopy and were pooled to a total of 500 g to make a composite sample per tree. Likewise, fully expanded leaf samples were taken from the four cardinal directions of the tree canopies to obtain a composite sample of leaves for each tree.

### Soil Chemical Analyses

Soil chemical analyses were conducted for the study reported in López-García et al. (2018). Soil samples were air-dried and sieved to <2 mm for chemical analysis. Soil pH, Ca, K, P, NH_4_, NO_3_, total C, total N and total TEs were measured, following the methodologies described in that paper. For this study, available concentrations of S and TEs were also analyzed. Sulfur and TEs were extracted from samples (<60 μm) with a 0.01 M CaCl_2_ solution (Houba et al., 2000) and analyzed by inductively coupled plasma spectrophotometry (ICP-OES) using a Varian ICP 720-ES (simultaneous ICP-OES with axially viewed plasma).

### Soil Enzyme Activities

The activity of three extracellular enzymes involved respectively in C, N, and P cycling [β-glucosidase (BGL), N-acetylglucosaminidase (NAG), and acid phosphatase (ACP)] were measured as indicators of microbial activity in the collected soils. These enzymes were analyzed colorimetrically by incubation with p-nitrophenyl-linked substrates at 37°C for 1 h, according to Tabatabai and Bremner (1969); Tabatabai (1982), Parham and Deng (2000), respective methods.

### ECM Species Composition and Functional Traits

Molecular analysis of ECM in root samples, as well as quantification of ECM functional traits (abundance of rhizomorphs, emanating hyphae, and melanin content) were conducted by López-García et al. (2018). Briefly, a composite sample of 28 root fragments per tree was obtained by selecting the seven longest root fragments in each of the four root subsamples collected from each tree. A random individual root tip per root fragment was photographed for posterior trait quantification (presence of emanating hyphae and rhizomorph and colorimetric estimation of melanization, see López-García et al., 2018, Appendix 1). Community weighted means (CWMs), i.e., the averaged value for these traits per tree, was calculated as the number of root tips exhibiting emanating hyphae or rhizomorphs divided by the total number of quantified root tips (Lepš et al., 2011). The color value was averaged between the 28 root tips of each tree for having an overall estimation of the ECM melanization of the community. The remaining material was used for the quantification of the percentage of root length colonized by ECM fungi, using the gridline intersect method (Brundrett et al., 1996; Navarro-Fernández et al., 2016). All these data was reported by López-García et al. (2018) and was included in the statistical analyses in order to evaluate the influence of ECM communities on holm oak status.

A small portion of each root tip was cut and immersed separately into 10 μl of Extraction Solution (Extract-N-Amp^TM^ Plant PCR Kit by Sigma-Aldrich) and the protocol of the manufacturer was followed to extract its DNA. PCR amplification was conducted using primers ITS1F (Gardes and Bruns, 1993) and ITS4 (White et al., 1990) following the procedure described in López-García et al. (2018), and Sanger sequenced. Sequences were blasted against the UNITE database (Kõljalg et al., 2013) and those found to correspond to ECM fungi were grouped by genera or family (see López-García et al., 2018 for details) and compared against the UNITE database (Kõljalg et al., 2013) for their taxonomic placement and Species Hypothesis determination. The number of root tips belonging to each root was used as abundance data.

### Plant Functional Traits

Root and leaf functional traits were measured specifically for this study, following the protocol described in Pérez-Harguindeguy et al. (2013). Morphological root traits included specific root length (SRL), specific root area (SRA), and root dry matter content (RDMC) and were measured with WinRHIZO 2009 (Regent Instruments, Quebec, CA, United States). Specific leaf area (SLA) and leaf dry matter content (LDMC) were measured in a subsample of 10 leaves per tree: leaves were scanned and analyzed with Image-Pro 4.5 (Media Cybernetic, Rockville, MD, United States).

After sampling, we selected 10 separated leaves from each tree and washed them with deionized water to determine the Chlorophyll Content Index (CCI) with a SPAD-502 chlorophyll meter (Minolta Camera, Co. Ltd., Osaka, Japan) taking three measurements per leaf.

Subsamples of roots and leaves collected from each tree were used for chemical analysis. This root material can be considered as the symbiotic combination of plant and fungi tissues. These subsamples were washed with distilled water, dried at 70°C for at least 48 h, and ground. Total C and N were determined by using a Flash 2000 HT elemental analyzer (Thermo Scientific, Bremen, Germany). Trace elements (As, Cd, Cu, Fe, Mn, Ni, Pb, and Zn) and macronutrients (S, P, K, Ca, and Mg) were determined by ICP-OES after digestion of plant tissues by wet oxidation with concentrated HNO_3_ in a Digiprep MS block digester (Domínguez et al., 2008).

### Data Analysis

In order to explore the relationships among ECM and plant variables we conducted a preliminary selection of key variables to be included in subsequent multivariate and modeling analyses. As the aim of the work was to evaluate whether plant performance (nutrient status and TE accumulation) is mediated by ECM communities in these soils, the subset of variables used as predictor variables included soil background properties and TEs, ECM species composition and ECM traits. The subset of response variables included TEs transfer from soil to root and leaves, root traits and leaf traits (Supplementary Figure 1). Soil variables and the characterization of ECM community, published by López-García et al., 2018, were used for the analysis of the present study.

A preliminary analysis of variance (ANOVA) to compare differences in soil, root, and leaf variables among sampling sites was performed. We checked for normality and homoscedasticity of data, and when assumptions were not met data were log or square root transformed. When these assumptions were met a Tukey’s Honest *post hoc* test followed. Otherwise, a non-parametric Kruskal–Wallis test and a Dunn’s test corrected by Bonferroni *post hoc* were performed.

Due to the dataset complexity, and in order to remove correlations and to reduce collinearity between soil variables, a principal component analysis (PCA) was performed to select a non-collinear subset of soil TEs to be used as predictors of plant traits in subsequent statistical analysis. Original data was log-transformed for normalization. Most correlated TEs with the first two axes of each PCA were selected for subsequent analyses.

In order to reduce ECM fungal species composition into two dimensions, a principal coordinate analysis (PCoA) was performed with the operational taxonomic units (OTUs) matrix (Legendre and Gallagher, 2001). The first two PCoA axes were selected (Supplementary Table 1) (Pinheiro and Bates, 2000; Zuur, 2009).

To evaluate the influence of soil and ECM variables on plant nutritional status and its functional traits we applied both correlational analysis and linear mixed models. In order to understand the relationships between the response and predictor variables, we first performed Pearson’s correlation tests, adjusted with Benjamini-Hochberg correction (Benjamini and Hochberg, 1995). Those soil and ECM variables showing a significant correlation with plant variables were considered as fixed effect factors in univariate linear mixed effect models, with sampling site as random factor. The significant variables from univariate models were included additively in multivariate models, however variance inflated factors (VIFs) were calculated and variables with VIF > 3 were removed to avoid collinear predictors (Zuur et al., 2010). Models were compared against a null model, assuming no influence of any of these predictors on plant variables. The best and most parsimonious predictive models were selected based on the Akaike information criterion corrected for small sample sizes (AICc; Burnham and Anderson, 2002). Selected models were fitted, and marginal and conditional R^2^ values were computed. Marginal R^2^ (R^2^_LMMm_) is the variance explained by fixed factors, while conditional R^2^ (R^2^_LMMc_) is variance explained by both fixed and random factors (Nakagawa and Schielzeth, 2013). Requirements for normality and homoscedasticity of residuals were fulfilled in all the selected models.

All statistical analyses were carried out using the R software v.3.3.2 (R Core Team, 2016), using packages *ggplot2* (Wickham, 2009), *MuMIn* (Barton, 2017), *nlme* (Pinheiro et al., 2016), *psych* (Revelle, 2017), and *vegan* (Oksanen et al., 2016).

## Results

### Soil Characterization

As reported by López-García et al. (2018), soil pH was significantly different among sites; sites 2 and 4 showed a significantly higher pH than site 3 and, specially, than site 1 (Supplementary Table 2). Available TEs levels decreased from Site 1 to Site 4. About soil nutrients, sites 1 and 3 showed significantly higher NH_4_, NO_3_, total N and organic C than sites 1 and 4. Calcium concentration was significantly higher at site 2 with respect to the other sites. Phosphorus contents were not significantly different among sites (Supplementary Table 2). All soil enzyme activities presented the highest activity at site 3 and NAG and ACP activities were found significantly lower at site 2 (Supplementary Table 2).

### Reduction of Trace Element and Community Composition Variables for Model Analysis

**FIGURE 1:**
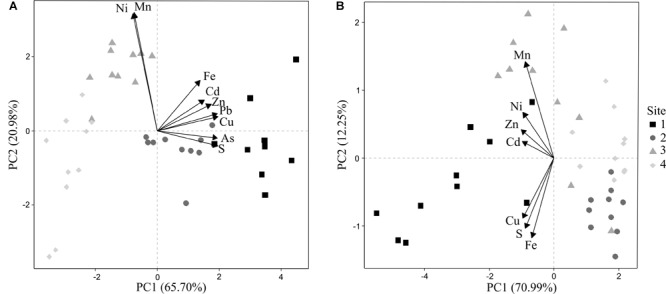
Principal component analysis (PCA) ordination of **(A)** soil total trace elements (TEs) and **(B)** soil available TEs, sampled at 0-20 cm depth under holm oak trees (*N* = 40) and classification by site.

Soil total TEs PC1 and PC2 ordination axis explained most of the total variance (86.68%) in the chemical composition of soils (Figure 1A). Axis 1 and 2 represented the variation of two clear groups of TEs which were orthogonal to each other. Axis 1 correlated well with total As, Cd, Cu, Pb, S and Zn, which tended to covariate. Axis 2 showed a high covariance between Mn and Ni. Likewise, the first two axes of available TEs explained most of the total variance (83.24%) (Figure 1B), being Zn and Mn the most correlated with axes PC1 and PC2 respectively. The final selected TEs included in the subsequent analyses were: total As, Fe and Mn, and available Mn and Zn concentrations. Available Cd was not chosen because some of the samples were below the detection limits. Lower guideline values (LGVs) for contaminated soils (Ministry of the Environment Finland, 2007) were exceeded for As, Cu, and Pb at site 1 (Supplementary Table 2).

**FIGURE 2:**
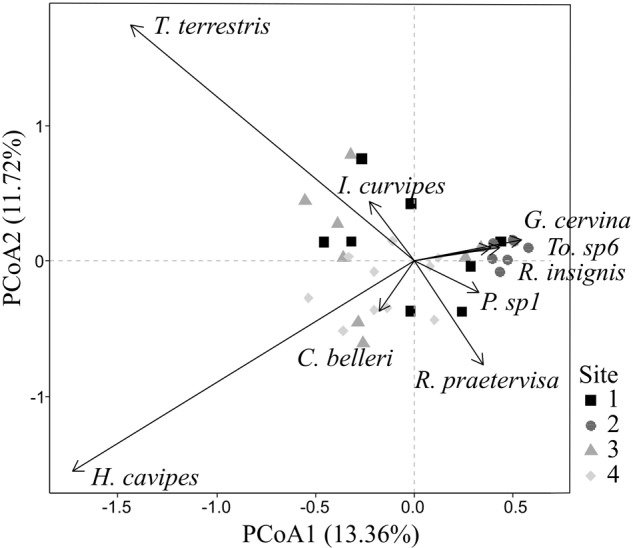
Principal coordinate analysis ordination of ECM fungal species in symbioses with holm oak roots and classification by site. Arrows indicate signicant ECM species (*p* < 0.05).

Fifty five OTUs were recorded belonging to ECM fungal species in 494 successfully sequenced root tips (published in López-García et al., 2018). In summary, these taxa comprised 14 families and 19 genera. The presence of rare species was common among the study: 19 of 55 OTUs were only identified in one root tip (Supplementary Figure 2). Two species, *Hebeloma cavipes* and *Thelephora terrestris* dominated the communities with 83 and 61 root tips, respectively (Supplementary Figure 2). The first two axes of PCoA of the ECM fungal communities explained a 25.08% of the variance in community composition. PCoA axis 1 (13.36%) showed a gradient from rare to abundant species (Figure 2). A clear pattern was also found in PCoA axis 2 (11.72% of explained variance) showing a transition of ECM fungal communities from *Thelephora terrestris* to *Hebeloma cavipes*.

### Relationships Between Soil and ECM Variables and Root Traits

In general, nutritional root status was found to be more affected by biotic factors than by abiotic ones when univariate models were run. Root C was the variable that was best explained by the considered predictors (Table 1). Soil Ca and available Mn (Estimate = 0.10) were those variables explaining the greatest variation in root C (univariate models), followed closely by melanization (Estimate = -0.23) (Table 1). Species composition PCoA1 (Estimate = -1.13), PCoA2 (Estimate = -1.14), and rhizomorph formation (Estimate = 0.08) also presented an effect on root C but to a lesser extent. Soil Ca content presented a negative effect on C root concentration. ECM species composition (PCoA1 and PCoA2 scores) was also related to root C; a higher root C was observed where *Hebeloma cavipes* species was dominant. In terms of biotic CWM traits, a low melanin content and high rhizomorph presence were also affecting root C (univariate models).

**Table 1:** Univariate and multivariate linear mixed models showing significant soil and ECM fungi fixed effects for each of the root traits and model explained variance.

Response variable	Individual effects of soil factors				Individual effects of ECM fungal factors				Combining significant effects into the best predictive model					
	Nutrients and EA		Trace elements		PCoA axis 1 and 2		Fungal traits CWM		Linear mixed effect models				Variance	
			
Root	Variable	*p*	Variable	*p*	Variable	*p*	Variable	*p*	Model	*SE*	*t*	*p*	R^2^_LMMm_	R^2^_LMMc_
C (%)	Ca	<0.001	Av. Mn	<0.001	PCoA1	0.048	Melanization	0.003	C = 45.43 – 0.001 Ca	0.0003	–4.11	<0.001	0.31	0.31
					PCoA2	0.044	Rhizomorph	0.047						
N (%)	–	–	Total Zn	0.006	(PCoA1)	(0.053)	–	–	N = 0.25 + 0.0006 Total Zn	0.0002	2.90	0.006	0.19	0.19
C:N	–	–	Total Zn	0.010	PCoA1	0.012	–	–	C:N = 141.70 – 29.64 PCoA1	11.07	–2.68	0.012	0.22	0.40
P (%)	–	–	–	–	PCoA1	0.009	–	–	P = 0.09 + 0.02 PCoA1	0.007	2.80	0.009	0.21	0.22
SRA (m^2^ kg^-1^)	–	–	–	–	PCoA1	0.045	–	–	SRA = 9.47 + 0.62 PCoA1	0.30	2.10	0.045	0.12	0.12
RDMC (mg g^-1^)	–	–	–	–	PCoA1	0.006	–	–	RDMC = 427.04 – 22.04 PCoA1	7.40	–2.98	0.006	0.22	0.22

*SRA, specific root area; RDMC, root dry matter content; Av., available; CWM, community weighted mean; EA, enzyme activity; SE, standard error; R^*2*^_*LMMm*_, marginal variance; R^*2*^_*LMMc*_, conditional variance.*

**FIGURE 3:**
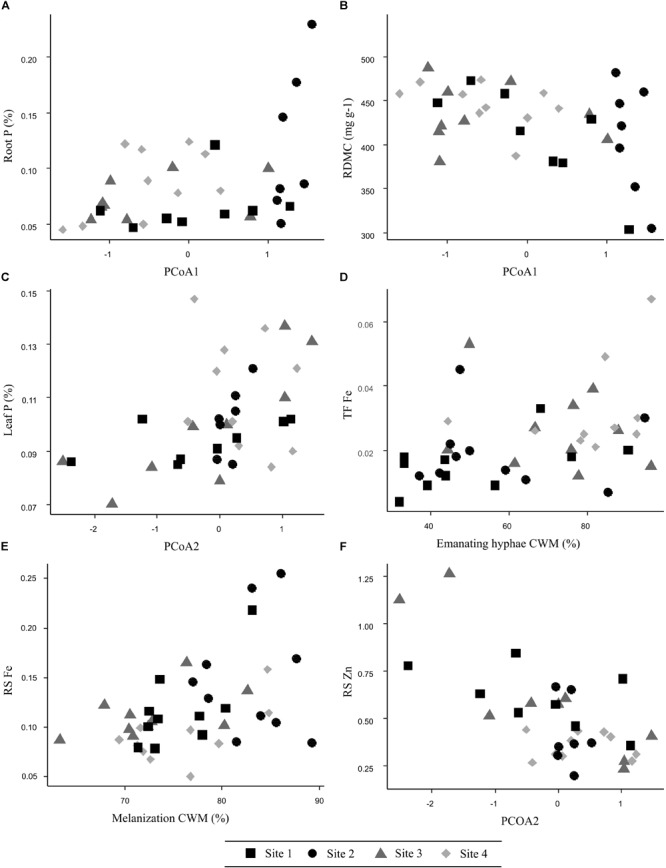
Relationship between selected key ECM fungal species composition and traits and their effects on **(A)** root P, **(B)** root dry matter content, **(C)** leaf P, **(D)** translocation factor Fe, **(E)** soil-to-root Fe transfer, and **(F)** soil-to-root Zn transfer in studied sites.

Root N was best explained by single total Zn, which exerted a positive effect (Table 1). In terms of biotic effects, species composition PCoA1 had a marginally significance influence (Estimate = 0.05). Root C:N ratio corroborated the role of total Zn on root N, as Zn was found negatively correlated to C:N ratio. The best model for root C:N included species composition PCoA1, exerting a negative effect, which confirmed that the presence of the most abundant species, *Hebeloma cavipes* and *Thelephora terrestris*, negatively influenced root N content. Root P was not explained by any abiotic factor but was positively affected by ECM species composition PCoA1 (Figure 3A and Table 1). As well as root N, root P was found to be lower when *Hebeloma cavipes* and *Thelephora terrestris* species were abundant in roots, therefore the symbioses with other species, here considered as rare due to their lower abundance, probably improved the nutritional status of holm oak roots in terms of P. Root N:P ratio was not significantly explained by any abiotic or biotic factor.

Both morphological root traits, SRA and RDMC were better explained by species composition PCoA1 than by any abiotic factors, but their effects were opposite (Table 1). The presence of the most abundant species, *Hebeloma cavipes* and *Thelephora terrestris* reduced SRA but increased RDMC (Figure 3B), while SRL was not significantly explained by any of the measured soil or ECM fungal factors. In summary, a key effect of species composition was found for those variables related to root nutrition variables. The abundance of *Thelephora terrestris* and, in special, *Hebeloma cavipes* species seemed to be related to high C, C:N ratio and RDMC values, and low N, P and SRA values in holm oak roots.

Marginal and conditional R^2^ for all the response variables, except C:N ratio, were similar. Variance explained by conditional R^2^ for the C:N ratio response almost doubled the marginal R^2^ (Table 1).

### Relationships Between Soil and ECM Variables and Transfer of Trace Elements to Roots

Transfer of TEs from soil to root seemed to be mainly driven by biotic factors: species composition PCoA2 and melanization CWM (Table 2). The soil-to-root transfer of As (RS As) was related to species composition PCoA2 and ECM melanization (Table 2). A high abundance of *Hebeloma cavipes* species and high melanin content seemed to be associated to a high As transfer to roots. A negative relationship with soil As was also found (Estimate = -0.0004). The soil-to-root transfer of Fe (RS Fe) was positively explained by soil NO_3_ and melanization (Figure 3E and Table 2). Species composition PCoA1 was also positively related (Estimate = 0.026), meaning that in those soils where rare species were abundant, Fe transfer to roots was higher. Rhizomorphs formation was negatively related (Estimate = -0.01). The soil-to-root transfer of Mn (RS Mn) was best explained by abiotic variables, namely soil Ca and soil Mn, which were negatively associated with this transfer (Table 2). Species composition PCoA2 showed an individual negative effect on Mn transfer (Estimate = -0.06), therefore the abundance of *Hebeloma cavipes* species in the soil was found to be positively related to Mn transfer from soils to roots. The soil-to-root transfer of Zn (RS Zn) was negatively affected by species composition PCoA2 (Figure 3F and Table 2). Therefore, as previously found for As, a higher abundance of *Hebeloma cavipes* species increased the soil-to-root transfer of Zn. In this case, the significant effect of melanization was negative (Estimate = -0.01), opposite to the As and Fe transfers. Soil Ca (Estimate = -0.00009) showed an individual negative effect on soil-to-root transfer of Zn.

**Table 2:** Univariate and multivariate linear mixed models showing significant soil and ECM fungi fixed effects for each of the soil-to-root (RS) transfer and translocation factor (TF) and model explained variance.

Response variable	Individual effects of soil factors				Individual effects of ECM fungal factors				Combining significant effects into the best predictive model					
	Nutrients and EA		Trace elements		PCoA axis 1 and 2		Fungal traits CWM		Linear mixed effect models				Variance	
			
Transfer	Variable	*p*	Variable	*p*	Variable	*p*	Variable	*p*	Model	*SE*	*t*	*p*	R^2^_LMMm_	R^2^_LMMc_
RS As	–	–	Total As	0.036	PCoA2	0.008	Melanization	0.050	RS As = -0.32 - 0.04 PCoA2	PCoA2 0.01	PCoA2 -3.54	PCoA2 0.001	0.27	0.63
									+ 0.006 Melanization	Mel 0.002	Mel 2.90	Mel 0.007		
RS Fe	NO_3_	<0.001	–	–	PCoA1	0.002	Melanization	0.004	RS Fe = -0.12 + 0.009 NO_3_	NO_3_ 0.002	NO_3_ 4.39	NO_3_ < 0.001	0.48	0.51
							Rhizomorph	0.021	+ 0.003 Melanization	Mel 0.0009	Mel 2.99	Mel 0.005		
RS Mn	Ca	0.003	Total Mn	0.004	PCoA2	0.039	–	–	RS Mn = 0.61 - 0.00005 Ca -0.0003 Total Mn	Ca 0.00001 Total Mn 0.00009	Ca -4.09 Total Mn -3.83	Ca < 0.001 Total Mn < 0.001	0.44	0.44
RS Zn	Ca	<0.001	–	–	PCoA2	<0.001	Melanization	0.041	RS Zn = 0.50 - 0.16 PCoA2	0.03	–5.29	<0.001	0.46	0.51
TF Fe	–	–	–	–	–	–	Hyphae	0.026	TF Fe = 0.009 + 0.0002 Hyphae	0.0001	2.32	0.026	0.13	0.13
TF Mn	NAG	<0.001	–	–	PCoA1	0.004	–	–	TF Mn = 3.62 + 1.82 PCoA1	0.58	3.15	0.004	0.16	0.75
TF Zn	–	–	Total Zn	0.007	–	–	–	–	TF Zn = 0.98 - 0.002 Total Zn	0.0007	–2.88	0.007	0.22	0.61

*NAG, *N*-acetyl-glucosaminidase; CWM, community weighted mean; EA, enzyme activity; SE, standard error; R^2^_LMMm_, marginal variance; R^2^_LMMc_, conditional variance.*

Marginal and conditional R^2^ showed similar percentage of variances for Fe, Mn, and Zn transfer but transfer of As was more explained by the site random effect (conditional R^2^) than the biotic fixed effects (Table 2).

### Relations of Soil Factors With Translocation of Trace Elements to Leaves

Translocation of TEs from roots to leaves were explained by different abiotic and biotic factors (Table 2), depending on the element. Translocation of As was not significantly explained by any individual abiotic or biotic factor. Due to the non-significant fixed effect of the model for the response variable translocation factor of As, a covariate Cu transfer was studied. Translocation factor of Cu was highly explained by soil Cu and species composition PCoA2 (R^2^_LMMm_ = 0.55; R^2^_LMMc_ = 0.76). Soil Cu contamination showed a significant negative effect on the Cu translocation (*p* < 0.001) while PCoA2 showed a significant positive effect (*p* = 0.013), therefore Cu translocation was favored on *Thelephora terrestris* dominated soils. Iron translocation from roots to leaves was only significantly explained by the biotic emanating hyphae, showing a positive relationship (Figure 3D and Table 2). Translocation factor of Mn was significantly related to NAG enzyme activity and species composition PCoA1, being this last variable the most explicative, showing a positive effect (Estimate = 8.54) (Table 2). Translocation factor of Zn was only significantly explained by soil Zn, however, Zn showed a negative effect on Zn transfer (Table 2).

High differences between marginal and conditional R^2^ variance were found for all TE translocation factors, except for Fe (Table 2).

### Relations of Soil Factors With Leaf Traits

Nutritional status of holm oak leaves were, in general, highly affected by soil P and ECM fungal species composition (PCoA1 and PCoA2 factors; Table 3). Leaf C was highly explained by a combination of abiotic and biotic factors (soil As and species composition PCoA1 factors, Table 3). Both predictor variables showed a strong positive relationship with leaf C. Emanating hyphae was also found to influence leaf C content, but negatively (Estimate = -0.09), when univariate relationships were analyzed. Leaf N was significantly influenced by soil P (Table 3) which explained a high proportion of variance of leaf N. No biotic factor was identified as significant for leaf N. Leaf C:N ratio was also highly explained by soil P but a negative effect was observed, in coherence with leaf N effects. A biotic effect was significantly found in relation to species composition PCoA1. The positive effect (Estimate = 2.04) of PCoA1 on this ratio showed consistency with model effects on leaf C. In summary, the results from these models showed a higher leaf C content and, therefore a higher C:N ratio, in those sites with particular abiotic characteristics (high As contamination and low soil P), and associated with certain biotic features: low abundance of *Hebeloma cavipes* and *Thelephora terrestris*, and low emanating hyphae.

**Table 3:** Univariate and multivariate linear mixed models showing significant soil and ECM fungi fixed effects for each of the leaf traits and model explained variance.

Response variable	Individual effects of soil factors				Individual effects of ECM fungal factors				Combining significant effects into the best predictive model					
	Nutrients and EA		Trace elements		PCoA axis 1 and 2		Fungal traits CWM		Linear mixed effect models				Variance	
			
Leaf	Variable	*p*	Variable	*p*	Variable	*p*	Variable	*p*	Model	*SE*	*t*	*p*	R^2^_LMMm_	R^2^_LMMc_
C (%)	–	–	Total As	0.014	PCoA1	0.010	Hyphae	<0.001	C = 48.56 + 1.93 PCoA1 + 0.019 Total As	PCoA1 0.50 Total As 0.006	PCoA1 3.86 Total As 2.87	PCoA1 < 0.001 Total As 0.008	0.51	0.51
N (%)	P	<0.001	–	–	–	–	–	–	N = 1.20 + 0.001 P	0.002	4.45	<0.001	0.35	0.35
C:N	P	<0.001	–	–	PCoA1	0.048	–	–	C:N = 42.10 – 0.32 P	0.09	–3.47	<0.001	0.25	0.26
P (%)	P	0.035	Av. Mn	0.007	PCoA2	0.007	–	–	P = 0.10 + 0.009 PCoA2	0.003	2.91	0.007	0.21	0.21
N:P	–	–	–	–	PCoA2	0.039	–	–	N:P = 13.30 – 0.90 PCoA2	0.42	–2.16	0.039	0.13	0.13
SLA (m^2^ kg^-1^)	NAG	0.002	–	–	–	–	–	–	SLA = 4.38 + 1.10 NAG	0.33	3.35	0.002	0.24	0.24
CCI (SPAD)	P	<0.001	–	–	–	–	–	–	CCI = 44.37 + 0.30 P	0.07	4.38	<0.001	0.30	0.49

*SLA, specific leaf area; CCI, Chlorophyll Content Index; NAG, *N*-acetyl-glucosaminidase; Av., available; CWM, community weighted mean; EA, enzyme activity; SE, standard error; R^*2*^_*LMMm*_, marginal variance; R^*2*^_*LMMc*_, conditional variance.*

Leaf P was best explained by species composition PCoA2 alone, which had a positive effect on this response variable (Figure 3C and Table 3). Soil P and available Mn had also a significant influence on leaf P, according to abiotic univariate models; soil P had a positive effect (Estimate = 0.0007) while Mn availability showed a negative effect (Estimate = -0.0005) on leaf P. Leaf N:P ratio was best predicted by species composition PCoA2 alone; the negative effect between PCoA2 and this the ratio corroborates the previous leaf P results. No abiotic variables were found to have a significant effect on leaf N:P ratio. To summarize, a higher leaf P and a lower N:P ratio were found in soils with high P, low Mn availability and dominance of *Thelephora terrestris* over *Hebeloma cavipes* species.

Morphological trait SLA was best related with soil NAG enzyme activity, while CCI was significantly related to soil P. For both SLA and CCI no mycorrhizal variables were significant predictors of their variance (Table 3). In addition, no significant variables were found to explain LDMC variation.

Marginal and conditional R^2^ for all the leaf response variables were akin except for CCI which presented a higher conditional variance (Table 3).

## Discussion

In this study we aimed to quantify the influence of ECM fungal communities on certain plant morphological and chemical traits, and to assess whether they may influence host status. The scenario chosen for this purpose was a TE contaminated area in which the effect of the abiotic factors, including the TE contamination and the soil background variables, on the community composition and functional traits of ECM fungi had been already tested (López-García et al., 2018). Hence, since the abiotic environment was indeed shaping the ECM communities, any effect of the latter on plant traits must be interpreted as a mediated effect of the ECM fungi on soil–plant relationships. In general, we found that ECM community composition and traits explained more than the abiotic environment for most of the measured plant traits.

### Root Functional Traits

Root systems are known to show a high plasticity in their development depending on soil local heterogeneity (Ostonen et al., 2007). In this study, we found several significant relationships between soil variables and root traits in holm oak trees with similar age and origin, which suggests high root plasticity in response to the studied environmental gradient. We further found that root functional traits were highly explained by the ECM community (in terms of both fungal species and traits), which corroborates the important mediation role of ECM on plant status and performance, and the need of incorporating symbiotic traits into the analysis of root traits (Weemstra et al., 2016).

In relation to the root economics spectrum, we could align the presence of abundant species of ECM (*Hebeloma cavipes* and *Thelephora terrestris*) with conservative positions into the root economics spectrum, i.e., exhibiting conservative traits such as a high C:N ratio and a low N and P content, and consequently a high C content, high RDMC and low SRL (de la Riva et al., 2016, 2018). The basidiomycete *Thelephora terrestris* is a common symbiotic ECM fungus (Marx et al., 1984; Menkis and Vasaitis, 2011) with beneficial effects for trees growing under stressful conditions, such as those that prevail in mine areas and reclamation sites (Lee and Koo, 1983), given that it protects the host by decreasing metal (Cu) transfer from soil to roots (Van Tichelen et al., 2001). Although *Hebeloma* spp. have been frequently found in heavy-metal contaminated soils (Colpaert et al., 2011) the abundant *Hebeloma cavipes* taxa is associated in the study area with soils with a low level of TE contamination (López-García et al., 2018). In terms of ECM traits, a high rhizomorph formation and low melanin content characterized those ECM fungi (i.e., *Thelephora terrestris* and *Hebeloma cavipes*) that were colonizing roots showing the most conservative traits. The presence of rhizomorphs, which functionally increases water and phosphate uptake through a long-distance exploration mechanism (Agerer, 2001), may be a consequence of resource limitations, hence constituting a conservative trait. Although melanin plays a role in protecting the root cells against high concentrations of heavy metals in the soil (Gadd and de Rome, 1988) these ECM fungi may prevent toxicity with other mechanisms (Bellion et al., 2006).

In the opposite edge of the root economics spectrum, we found roots colonized mostly by rare species and showing more acquisitive features, i.e., a high N and P concentration, a low RDMC and high SRA (de la Riva et al., 2016, 2018). These root traits might be indicating less resource limitations, probably due to higher soil nutrient contents and thus less dependency on rhizomorphs for nutrient acquisition. The fact that these roots belong to trees growing in soils with a high level of TE contamination could explain the higher degree of melanization of these fungi, in order to avoid TE toxicity.

In this study, we might have anticipated that the adverse soil chemical conditions posed by the contamination episode could have modulated root acquisition strategies, with roots growing in the most contaminated soils showing a more conservative strategy. However, conservative root traits were related to low soil TE (Zn) concentrations. On one hand, it is possible that the *a priori* concern about TE contamination as the main factor of stress for plant performance is masked by other sources of stress, such as water or nutrient limitations. Recently, López-García et al. (2018) found that soil background properties and TEs concentrations explained the same proportion of variance in ECM species composition, which support this concept. On the other hand, although the root economics spectrum is associated to nutrient absorption and soil fertility, here we found that other factors such as ECM community composition and TE contamination could support the multidimensional root trait framework. Mycorrhizal fungi have a fundamental role in acquiring resources but also protecting plants from the negative impact of some sources of biotic and abiotic stress. Trace element contamination seems to be independent from root economics spectrum, which indicates the existence of a multidimensional framework that includes other processes different from those related to nutrient uptake (Weemstra et al., 2016).

### Soil-Root-Leaf Transfer of Trace Elements

Trace element mobility through the soil-root-leaf continuum depends on several factors, and obviously initial concentrations in the soil is one of them (Kabata-Pendias, 2004). Despite that the range of soil TE concentrations in our environmental gradient was relatively large (for example, total As concentrations ranged from 6.83 to 286 mg kg^-1^), accumulation of TEs in oak leaves was relatively low, and leaf TE concentrations were within the normal ranges (except Mn levels; over 400 mg kg^-1^) and below the levels that can be toxic to plants (Madejón et al., 2002). This confirms that holm oak is a suitable species for the phytostabilization of contaminated soils, given its ability to prevent TE accumulation into aboveground biomass (Supplementary Table 2). Previous work under controlled greenhouse conditions showed that this species has a capacity to retain and tolerate high concentrations of some TEs (Cd) into fine roots (Domínguez et al., 2009). Mechanisms involved in TE retention into the root system include adsorption onto roots, or precipitation within the rhizosphere (Pulford and Watson, 2003; Wong, 2003). The pectin in the cell wall are the main constituents allowing metal binding due to their carboxyl groups, which have a high cation exchange capacity (Franco et al., 2002). In the present study, the soil-to-root transfer of As, Mn, and Zn was highly explained by ECM fungal species composition and traits, which suggests that interactions with fungi play an important role at determining the capacity of this species to retain TEs into its roots. The highest transfer of these TEs from soil to roots (plant and fungi tissue) was observed in trees whose roots were colonized by *Hebeloma cavipes*. In contrast, soil-to-root transfer of Fe presented a different trend, with the highest transfer being recorded in roots colonized by rare species taxa. This confirms that the mechanisms by which mycorrhizal fungi participate in metal uptake by plants can differ for each element and each fungal species (Godbold et al., 1998; Jentschke and Godbold, 2000).

Melanization was corroborated as a trait with a role in the protection of plants against heavy metals (Gadd and de Rome, 1988), as it was highly positively related to TE transfer to roots, although the relationship between melanin content and transfer of TEs from soils to roots differed across elements. Melanization was positively related to As and Mn but negatively related to Zn transfer. These opposite trends could indicate that roots are subjected to multiple constraints (Weemstra et al., 2016) in these multi-metal contaminated soils, and that different elements affect differently to these ECM traits.

### Leaf Functional Traits

Resource availability directly impacts functional traits such as SLA and leaf N and P content (Friesen et al., 2011). It was expected that ECM fungal mediation would increase resource acquisition by plants by accessing to organic forms unavailable to plants and by more efficient foraging (Friesen et al., 2011).

Leaf C is captured via photosynthesis, therefore C uptake is not mediated by ECM fungi. But assimilation of C into plant tissue might be affected by a range of factors, such as nutritional status and water stress, in which ECM community may play certain role (Cornelissen et al., 2001), as explored here. A high positive relationship was found between ECM species composition (mainly, in relation to the presence of rare species) and leaf C and C:N ratio; that could be an indirect effect of ECM community composition through its effects on root functional traits. Leaf P and N:P ratio were related to ECM species composition as well, specifically high leaf P was related to the root colonization by *Thelephora terrestris.* This is in agreement with Van Tichelen et al. (2001), who showed that *Thelephora terrestris* played a central role in the P nutrition of the host plant in a P-limited and Cu-contaminated soil.

Plants are performing a continuous carbon and nutrient investment in order to maintain the key leaf functions (i.e., photosynthesis) (Poorter and Bongers, 2006). Leaf N is responsible of the photosynthetic machinery, especially Rubisco, and leaf P is found in nucleic acids, lipid membranes and bioenergetic molecules (Wright et al., 2004), therefore both are key chemical traits. Leaf N correlated positively with CCI and this result agreed with that an optimal leaf N is essential for photosynthesis (de la Riva et al., 2016). Leaf N, P and CCI have shown a high positive relationship to soil P. A phosphorus limitation in soils has been previously registered in the study area (Domínguez et al., 2010) and it is known that leaf nutrient traits are more closely linked to soil P under limiting conditions (Niinemets and Kull, 2003; Liu et al., 2010; Chen et al., 2011).

The leaf traits SLA and CCI were not related to ECM fungal species composition or traits. These functional traits are related to light capturing functions (Niinemets and Sack, 2006) which here have been found not to be mediated by ECM, but affected by soil variables (i.e., NAG and P).

### Feedback Effect of the Symbiosis ECM Fungi–Host Plant

Pollution by TEs may favored the dominance of tolerant ECM fungal species, altering the ECM fungal composition (Hui et al., 2011; Op De Beeck et al., 2015). Abundant *Thelephora* and *Hebeloma* taxa have been previously found in areas contaminated by different TEs such as Cd, Cu, Mn, Pb, or Zn (Hartley et al., 1997; Van Tichelen et al., 2001; Hui et al., 2011; Huang et al., 2014; De Oliveira and Tibbett, 2018). Therefore, there may be a selection of these ECM species which are able to tolerate TE contamination probably through extracellular and intracellular mechanisms (Jentschke and Godbold, 2000; Bellion et al., 2006; Ciadamidaro et al., 2017). These ECM species would protect the host plant by decreasing TE transfer and shaping plant functional traits (Van Tichelen et al., 2001; Bauman et al., 2016). Although this study has not studied how plant communities and their traits are also responsible of structuring ECM communities composition, previous studies (de Vries et al., 2012; Aponte et al., 2013; Bauman et al., 2016; López-García et al., 2017) have found the existence of feedback processes. Due to the ecological complexity of the soil–plant interaction system, further research is needed to understand the ECM fungi and host plant relevant traits, as well as genetic variation, which allow the establishment of the host plant in TE contaminated soils. Finally, a better understanding of the symbiosis would improve the planning and outcomes of phyto- and myco-remediation strategies (Ali et al., 2017; Ciadamidaro et al., 2017).

## Conclusion

The analysis of root and leaf traits, as well as ECM communities and soil physico-chemical properties in a large-scale phytoremediated area, revealed that plant functions, expressed as variations in plant traits, can be affected in similar extents by the abiotic and the biotic environment that surround and interact with each individual plant. We could identify some ECM fungal community traits that were highly related to the studied plant variables (root traits, nutrient status, and TE accumulation), in a greater extent than the abiotic environment. In some cases, such as the transfer of As, Mn and Zn, the best explanatory variable was directly related to the composition of the ECM community, suggesting species-specific mechanisms of interactions between holm oak and ECM fungi. ECM traits co-varied with the root economics spectrum, as ECM rhizomorphs and melanization traits were related to the acquisitive-conservative root spectrum. Future studies on plant–soil interactions in contaminated soils should therefore consider that critical processes, such as nutrient assimilation and TE accumulation into biomass, can be largely mediated by ECM fungi.

## Author Contributions

MG-M, ÁL-G, CN-F, MD, and TM designed the study and conducted the sampling. MG-M, ÁL-G, CN-F, MD, and RK conducted the laboratory analyses. MG-M, ÁL-G, and MD conducted the data analyses. MG-M wrote the manuscript with contributions from all the authors.

## Conflict of Interest Statement

The authors declare that the research was conducted in the absence of any commercial or financial relationships that could be construed as a potential conflict of interest.

## References

[B1] Agerer R. (2001). Exploration types of ectomycorrhizae: a proposal to classify ectomycorrhizal mycelial systems according to their patterns of differentiation and putative ecological importance. *Mycorrhiza* 11 107–114. 10.1007/s005720100108

[B2] Ali A. Guo D. Mahar A. Wang P. Shen F. Li R. (2017). Mycoremediation of potentially toxic trace elements—a biological tool for soil cleanup: a review. *Pedosphere* 27 205–222. 10.1016/S1002-0160(17)60311-4

[B3] Aponte C. García L. V. Marañón T. (2013). Tree species effects on nutrient cycling and soil biota: a feedback mechanism favouring species coexistence. *For. Ecol. Manage.* 309 36–46. 10.1016/j.foreco.2013.05.035

[B4] Aponte C. García L. V. Marañón T. Gardes M. (2010). Indirect host effect on ectomycorrhizal fungi: leaf fall and litter quality explain changes in fungal communities on the roots of co-occurring Mediterranean oaks. *Soil Biol. Biochem.* 42 788–796. 10.1016/j.soilbio.2010.01.014

[B5] Bardgett R. D. Mommer L. De Vries F. T. (2014). Going underground: root traits as drivers of ecosystem processes. *Trends Ecol. Evol.* 29 692–699. 10.1016/j.tree.2014.10.006 25459399

[B6] Barton K. (2017). *MuMIn: Multi-Model Inference.* Available at: https://cran.r-project.org/package=MuMIn

[B7] Bauman D. Raspé O. Meerts P. Degreef J. Muledi J. I. Drouet T. (2016). Multiscale assemblage of an ectomycorrhizal fungal community: the influence of host functional traits and soil properties in a 10-ha miombo forest. *FEMS Microbiol. Ecol.* 92:fiw151. 10.1093/femsec/fiw151 27402715

[B8] Bellion M. Courbot M. Jacob C. Blaudez D. Chalot M. (2006). Extracellular and cellular mechanisms sustaining metal tolerance in ectomycorrhizal fungi. *FEMS Microbiol. Lett.* 254 173–181. 10.1111/j.1574-6968.2005.00044.x 16445743

[B9] Benjamini Y. Hochberg Y. (1995). Controlling the false discovery rate?: a practical and powerful approach to multiple testing. *J. R. Stat. Soc. Ser. B* 57 289–300.

[B10] Bever J. D. (2003). Soil community feedback and the coexistence of competitors: conceptual frameworks and empirical tests. *New Phytol.* 157 465–473. 10.1046/j.1469-8137.2003.00714.x 33873396

[B11] Bever J. D. Dickie I. A. Facelli E. Facelli J. M. Klironomos J. Moora M. (2010). Rooting theories of plant community ecology in microbial interactions. *Trends Ecol. Evol.* 25 468–478. 10.1016/j.tree.2010.05.004 20557974 PMC2921684

[B12] Bolan N. S. Park J. H. Robinson B. Naidu R. Huh K. Y. (2011). Phytostabilization. A green approach to contaminant containment. *Adv. Agron.* 112 145–204. 10.1016/B978-0-12-385538-1.00004-4

[B13] Brinkman E. P. Van der Putten W. H. Bakker E. J. Verhoeven K. J. F. (2010). Plant-soil feedback: experimental approaches, statistical analyses and ecological interpretations. *J. Ecol.* 98 1063–1073. 10.1111/j.1365-2745.2010.01695.x

[B14] Brundrett M. Bougher N. Dell B. Grove T. Malajczuk N. (1996). Working with mycorrhizas in forestry and agriculture. ACIAR Monograph. *J. Biol. Chem.* 32:374 10.1046/j.1469-8137.1997.00703-7.x

[B15] Burnham K. P. Anderson D. R. (2002). *Model Selection and Multimodel Inference: A Practical Information-Theoretic Approach*, 2nd Edn. New York, NY: Springer. 10.1016/j.ecolmodel.2003.11.004

[B16] Cabrera F. Clemente L. Díaz Barrientos E. López R. Murillo J. M. (1999). Heavy metal pollution of soils affected by the Guadiamar toxic flood. *Sci. Total Environ.* 242 117–129. 10.1016/S0048-9697(99)00379-4 10635579

[B17] Chen F.-S. Niklas K. J. Zeng D.-H. (2011). Important foliar traits depend on species-grouping: analysis of a remnant temperate forest at the Keerqin Sandy Lands, China. *Plant Soil* 340 337–345. 10.1007/s11104-010-0606-9

[B18] Chen W. Koide R. T. Eissenstat D. M. (2018). Nutrient foraging by mycorrhizas: from species functional traits to ecosystem processes. *Funct. Ecol.* 32 858–869. 10.1111/1365-2435.13041 25970701

[B19] Ciadamidaro L. Girardclos O. Bert V. Zappelini C. Yung L. Foulon J. (2017). Poplar biomass production at phytomanagement sites is significantly enhanced by mycorrhizal inoculation. *Environ. Exp. Bot.* 139 48–56. 10.1016/j.envexpbot.2017.04.004

[B20] Colpaert J. V. Wevers J. H. L. Krznaric E. Adriaensen K. (2011). How metal-tolerant ecotypes of ectomycorrhizal fungi protect plants from heavy metal pollution. *Ann. For. Sci.* 68 17–24. 10.1007/s13595-010-0003-9

[B21] Cornelissen J. Aerts R. Cerabolini B. Werger M. Van der Heijden M. (2001). Carbon cycling traits of plant species are linked with mycorrhizal strategy. *Oecologia* 129 611–619. 10.1007/s004420100752 24577702

[B22] Crowther T. W. Maynard D. S. Crowther T. R. Peccia J. Smith J. R. Bradford M. A. (2014). Untangling the fungal niche: the trait-based approach. *Front. Microbiol.* 5:579. 10.3389/fmicb.2014.00579 25400630 PMC4215788

[B23] de la Riva E. G. Marañón T. Pérez-Ramos I. M. Navarro-Fernández C. M. Olmo M. Villar R. (2018). Root traits across environmental gradients in Mediterranean woody communities: are they aligned along the root economics spectrum? *Plant Soil* 424 31–48. 10.1007/s11104-017-3433-4

[B24] de la Riva E. G. Tosto A. Pérez-Ramos I. M. Navarro-Fernández C. M. Olmo M. Anten N. P. R. (2016). A plant economics spectrum in Mediterranean forests along environmental gradients: is there coordination among leaf, stem and root traits? *J. Veg. Sci.* 27 187–199. 10.1111/jvs.12341

[B25] De Oliveira V. H. Tibbett M. (2018). Cd and Zn interactions and toxicity in ectomycorrhizal basidiomycetes in axenic culture. *PeerJ* 6:e4478. 10.7717/peerj.4478 29568708 PMC5845391

[B26] de Vries F. T. Manning P. Tallowin J. R. B. Mortimer S. R. Pilgrim E. S. Harrison K. A. (2012). Abiotic drivers and plant traits explain landscape-scale patterns in soil microbial communities. *Ecol. Lett.* 15 1230–1239. 10.1111/j.1461-0248.2012.01844.x 22882451

[B27] Díaz S. Lavorel S. de Bello F. Quétier F. Grigulis K. Robson T. M. (2007). Incorporating plant functional diversity effects in ecosystem service assessments. *Proc. Natl. Acad. Sci. U.S.A.* 104 20684–20689. 10.1073/pnas.0704716104 18093933 PMC2410063

[B28] Domínguez M. T. Alegre J. M. Madejón P. Madejón E. Burgos P. Cabrera F. (2016). River banks and channels as hotspots of soil pollution after large-scale remediation of a river basin. *Geoderma* 261 133–140. 10.1016/j.geoderma.2015.07.008

[B29] Domínguez M. T. Madrid F. Marañón T. Murillo J. M. (2009). Cadmium availability in soil and retention in oak roots: potential for phytostabilization. *Chemosphere* 76 480–486. 10.1016/j.chemosphere.2009.03.026 19375778

[B30] Domínguez M. T. Marañón T. Murillo J. M. Schulin R. Robinson B. H. (2008). Trace element accumulation in woody plants of the Guadiamar Valley, SW Spain: a large-scale phytomanagement case study. *Environ. Pollut.* 152 50–59. 10.1016/j.envpol.2007.05.021 17602809

[B31] Domínguez M. T. Marañón T. Murillo J. M. Schulin R. Robinson B. H. (2010). Nutritional status of mediterranean trees growing in a contaminated and remediated area. *Water Air Soil Pollut.* 205 305–321. 10.1007/s11270-009-0075-z

[B32] Eissenstat D. M. Kucharski J. M. Zadworny M. Adams T. S. Koide R. T. (2015). Linking root traits to nutrient foraging in arbuscular mycorrhizal trees in a temperate forest. *New Phytol.* 208 114–124. 10.1111/nph.13451 25970701

[B33] Erktan A. McCormack M. L. Roumet C. (2018). Frontiers in root ecology: recent advances and future challenges. *Plant Soil* 424 1–9. 10.1007/s11104-018-3618-5

[B34] Firmin S. Labidi S. Fontaine J. Laruelle F. Tisserant B. Nsanganwimana F. (2015). Arbuscular mycorrhizal fungal inoculation protects *Miscanthus*×*giganteus* against trace element toxicity in a highly metal-contaminated site. *Sci. Total Environ.* 52 91–99. 10.1016/j.scitotenv.2015.04.116 25958358

[B35] Franco C. R. Chagas A. P. Jorge R. A. (2002). Ion-exchange equilibria with aluminum pectinates. *Colloids Surf. A Physicochem. Eng. Asp.* 204 183–192. 10.1016/S0927-7757(01)01134-7

[B36] Friesen M. L. Porter S. S. Stark S. C. von Wettberg E. J. Sachs J. L. Martinez-Romero E. (2011). Microbially mediated plant functional traits. *Annu. Rev. Ecol. Evol. Syst.* 42 23–46. 10.1146/annurev-ecolsys-102710-145039

[B37] Gadd G. M. de Rome L. (1988). Biosorption of copper by fungal melanin. *Appl. Microbiol. Biotechnol.* 29 610–617. 10.1007/BF00260993 15993471

[B38] Gardes M. Bruns T. D. (1993). ITS primers with enhanced specificity for basidiomycetes—Application to identification of mycorrhizae and rusts. *Mol. Ecol.* 2 113–118. 10.1111/j.1365-294X.1993.tb00005.x 8180733

[B39] Garnier E. Navas M.-L. Grigulis K. (2016). *Plant Functional Diversity?: Organism Traits, Community Structure, and Ecosystem Properties.* Oxford: Oxford University Press 10.1111/aec.12498

[B40] Godbold D. L. Jentschke G. Winter S. Marschner P. (1998). Ectomycorrhizas and amelioration of metal stress in forest trees. *Chemosphere* 36 757–762. 10.1016/S0045-6535(97)10120-5

[B41] Hartley J. Cairney J. W. G. Meharg A. A. (1997). Do ectomycorrhizal fungi exhibit adaptive tolerance to potentially toxic metals in the environment? *Plant Soil* 189 303–319. 10.1023/A:1004255006170

[B42] Houba V. J. G. Temminghoff E. J. M. Gaikhorst G. A. van Vark W. (2000). Soil analysis procedures using 0.01 M calcium chloride as extraction reagent. *Commun. Soil Sci. Plant Anal.* 31 1299–1396. 10.1080/00103620009370514

[B43] Huang J. Nara K. Zong K. Wang J. Xue S. Peng K. (2014). Ectomycorrhizal fungal communities associated with Masson pine (*Pinus massoniana*) and white oak (*Quercus fabri*) in a manganese mining region in Hunan Province, China. *Fungal Ecol.* 9 1–10. 10.1016/j.funeco.2014.01.001

[B44] Hui N. Jumpponen A. Niskanen T. Liimatainen K. Jones K. L. Koivula T. (2011). EcM fungal community structure, but not diversity, altered in a Pb-contaminated shooting range in a boreal coniferous forest site in Southern Finland. *FEMS Microbiol. Ecol.* 76 121–132. 10.1111/j.1574-6941.2010.01038.x 21223331

[B45] Jentschke G. Godbold D. L. (2000). Metal toxicity and ectomycorrhizas. *Physiol. Plant.* 109 107–116. 10.1034/j.1399-3054.2000.100201.x 12651328

[B46] Kabata-Pendias A. (2004). Soil-plant transfer of trace elements - An environmental issue. *Geoderma* 122 143–149. 10.1016/j.geoderma.2004.01.004

[B47] Köhler J. Yang N. Pena R. Raghavan V. Polle A. Meier I. C. (2018). Ectomycorrhizal fungal diversity increases phosphorus uptake efficiency of European beech. *New Phytol.* 10.1111/nph.15208 [Epub ahead of print]. 29770963

[B48] Koide R. T. Fernandez C. Malcolm G. (2014). Determining place and process: functional traits of ectomycorrhizal fungi that affect both community structure and ecosystem function. *New Phytol.* 201 433–439. 10.1111/nph.12538 26207269

[B49] Kõljalg U. Nilsson R. H. Abarenkov K. Tedersoo L. Taylor A. F. S. Bahram M. (2013). Towards a unified paradigm for sequence-based identification of fungi. *Mol. Ecol.* 22 5271–5277. 10.1111/mec.12481 24112409

[B50] Kurm V. Van Der Putten W. H. Pineda A. Hol W. H. G. (2018). Soil microbial species loss affects plant biomass and survival of an introduced bacterial strain, but not inducible plant defences. *Ann. Bot.* 121 311–319. 10.1093/aob/mcx162 29329376 PMC5808785

[B51] Lee K. J. Koo C. D. (eds). (1983). “Inoculation of pines in a nursery with *Pisolithus tinctorius* and *Thelephora terrestris* in Korea,” in *Tree Root Systems and Their Mycorrhizas*, (Dordrecht: Springer), 325–329. 10.1007/978-94-009-6833-2_36

[B52] Legendre P. Gallagher E. D. (2001). Ecologically meaningful transformations for ordination of species data. *Oecologia* 129 271–280. 10.1007/s004420100716 28547606

[B53] Lepš J. de Bello F. Šmilauer P. Doležal J. (2011). Community trait response to environment: disentangling species turnover vs intraspecific trait variability effects. *Ecography* 34 856–863. 10.1111/j.1600-0587.2010.06904.x

[B54] Liu B. Li H. Zhu B. Koide R. T. Eissenstat D. M. Guo D. (2015). Complementarity in nutrient foraging strategies of absorptive fine roots and arbuscular mycorrhizal fungi across 14 coexisting subtropical tree species. *New Phytol.* 208 125–136. 10.1111/nph.13434 25925733

[B55] Liu G. Freschet G. T. Pan X. Cornelissen J. H. C. Li Y. Liu G. (2010). Coordinated variation in leaf and root traits across multiple spatial scales in Chinese semi-arid and arid ecosystems. *New Phytol.* 188 543–553. 10.1111/j.1469-8137.2010.03388.x 20649915

[B56] López-García Á. Gil-Martínez M. Navarro-Fernández C. M. Kjøller R. Azcón-Aguilar C. Domínguez M. T. (2018). Functional diversity of ectomycorrhizal fungal communities is reduced by trace element contamination. *Soil Biol. Biochem.* 121 202–211. 10.1016/j.soilbio.2018.03.021

[B57] López-García Á. Varela-Cervero S. Vasar M. Öpik M. Barea J. M. Azcón-Aguilar C. (2017). Plant traits determine the phylogenetic structure of arbuscular mycorrhizal fungal communities. *Mol. Ecol.* 26 6948–6959. 10.1111/mec.14403 29110362

[B58] Madejón P. Domínguez M. T. Gil-Martínez M. Navarro-Fernández C. M. Montiel-Rozas M. M. Madejón E. (2018a). Evaluation of amendment addition and tree planting as measures to remediate contaminated soils: the Guadiamar case study (SW Spain). *Catena* 166 34–43. 10.1016/j.catena.2018.03.016

[B59] Madejón P. Domínguez M. T. Madejón E. Cabrera F. Marañón T. Murillo J. M. (2018b). Soil-plant relationships and contamination by trace elements: a review of twenty years of experimentation and monitoring after the Aznalcóllar (SW Spain) mine accident. *Sci. Total Environ.* 625 50–63. 10.1016/j.scitotenv.2017.12.277 29289006

[B60] Madejón P. Murillo J. M. Marañón T. Cabrera F. López R. (2002). Bioaccumulation of As, Cd, Cu, Fe and Pb in wild grasses affected by the Aznalcóllar mine spill (SW Spain). *Sci. Total Environ.* 290 105–120. 10.1016/S0048-9697(01)01070-1 12083702

[B61] Marx D. H. Cordell C. E. Kenney D. S. Mexal J. G. Artman J. D. Riffle J. W. (1984). Commercial vegetative inoculum of *Pisolithus tinctorius* and inoculation techniques for development of ectomycorrhizae on bare-root tree seedlings. *For. Sci.* 30:a0001 10.1093/forestscience/30.s1.a0001

[B62] Mendez M. O. Maier R. M. (2008). Phytostabilization of mine tailings in arid and semiarid environments - An emerging remediation technology. *Environ. Health Perspect.* 116 278–283. 10.1289/ehp.10608 18335091 PMC2265025

[B63] Menkis A. Vasaitis R. (2011). Fungi in roots of nursery grown *Pinus sylvestris*: ectomycorrhizal colonisation, genetic diversity and spatial distribution. *Microb. Ecol.* 61 52–63. 10.1007/s00248-010-9676-8 20437259

[B64] Ministry of the Environment Finland (2007). *Government Decree on the Assessment of Soil Contamination and Remediation Needs (214/2007).* Available at: www.finlex.fi

[B65] Nakagawa S. Schielzeth H. (2013). A general and simple method for obtaining R2 from generalized linear mixed-effects models. *Methods Ecol. Evol.* 4 133–142. 10.1111/j.2041-210x.2012.00261.x

[B66] Navarro-Fernández C. M. Pérez-Ramos I. M. de la Riva E. G. Vera J. R. Roumet C. Villar R. (2016). Functional responses of Mediterranean plant communities to soil resource heterogeneity: a mycorrhizal trait-based approach. *J. Veg. Sci.* 27 1243–1253. 10.1111/jvs.12446

[B67] Niinemets Ü. Kull K. (2003). Leaf structure vs. nutrient relationships vary with soil conditions in temperate shrubs and trees. *Acta Oecol.* 24 209–219. 10.1016/S1146-609X(03)00094-8

[B68] Niinemets Ü. Sack L. (2006). Structural determinants of leaf light-harvesting capacity and photosynthetic potentials. *Prog. Bot.* 67 385–419. 10.1007/3-540-27998-9_17

[B69] Oksanen J. Blanchet F. G. Friendly M. Kindt R. Legendre P. McGlinn D. (2016). *vegan: Community Ecology Package.* Available at: https://cran.r-project.org/package=vegan

[B70] Op De Beeck M. Lievens B. Busschaert P. Rineau F. Smits M. Vangronsveld J. (2015). Impact of metal pollution on fungal diversity and community structures. *Environ. Microbiol.* 17 2035–2047. 10.1111/1462-2920.12547 24947496

[B71] Ostonen I. Püttsepp Ü. Biel C. Alberton O. Bakker M. R. Lõhmus K. (2007). Specific root length as an indicator of environmental change. *Plant Biosyst.* 141 426–442. 10.1080/11263500701626069

[B72] Parham J. A. Deng S. P. (2000). Detection, quantification and characterization of b - glucosaminidase activity in soil. *Soil Biol. Biochem.* 32 1183–1190. 10.1016/S0038-0717(00)00034-1

[B73] Pérez-Harguindeguy N. Díaz S. Garnier E. Lavorel S. Poorter H. Jaureguiberry P. (2013). New handbook for standardised measurement of plant functional traits worldwide. *Aust. J. Bot.* 61 167–234.

[B74] Pinheiro J. Bates D. DebRoy S. Sarkar D. R Core Team (2016). *nlme: Linear and Nonlinear Mixed Effects Models.* Available at: http://cran.r-project.org/package=nlme

[B75] Pinheiro J. C. Bates D. M. (eds) (2000). “Linear mixed-effects models: basic concepts and examples,” in *Mixed-Effects Models in S and S-PLUS* (New York, NY: Springer-Verlag) 3–56. 10.1007/0-387-22747-4_1

[B76] Poorter L. Bongers F. (2006). Leaf traits are good predictors of plant performance across 53 rain forest species. *Ecology* 87 1733–1743. 16922323 10.1890/0012-9658(2006)87[1733:ltagpo]2.0.co;2

[B77] Pulford I. D. Watson C. (2003). Phytoremediation of heavy metal-contaminated land by trees - A review. *Environ. Int.* 29 529–540. 10.1016/S0160-4120(02)00152-6 12705950

[B78] R Core Team (2016). *R: A Language and Environment for Statistical Computing.* Available at: http://www.r-project.org

[B79] Revelle W. (2017). *psych: Procedures for Personality and Psychological Research.* Available at: https://cran.r-project.org/package=psych

[B80] Rutten G. Gómez-Aparicio L. (2018). Plant-soil feedbacks and root responses of two Mediterranean oaks along a precipitation gradient. *Plant Soil* 424 221–231. 10.1007/s11104-018-3567-z

[B81] Tabatabai M. A. (1982). “Soil enzymes,” in *Methods of Soil Analysis. Part 2. Chemical and Microbiological Properties* eds Page A. L. Miller E. M. Keeney D. R. (Madison, WI: Soil Science Society of America) 903–947. 10.1016/0038-0717(69)90012-1

[B82] Tabatabai M. A. Bremner J. M. (1969). Use of p-nitrophenyl phosphate for assay of soil phosphatase activity. *Soil Biol. Biochem.* 1 301–307. 10.1016/0038-0717(69)90012-1

[B83] Tibbett M. Sanders F. E. (2002). Ectomycorrhizal symbiosis can enhance plant nutrition through improved access to discrete organic nutrient patches of high resource quality. *Ann. Bot.* 89 783–789. 10.1093/aob/mcf129 12102534 PMC4233838

[B84] van der Heijden M. G. A. Martin F. M. Selosse M.-A. Sanders I. R. (2015). Mycorrhizal ecology and evolution: the past, the present, and the future. *New Phytol.* 205 1406–1423. 10.1111/nph.13288 25639293

[B85] van der Heijden M. G. A. Scheublin T. R. (2007). Functional traits in mycorrhizal ecology: their use for predicting the impact of arbuscular mycorrhizal fungal communities on plant growth and ecosystem functioning. *New Phytol.* 174 244–250. 10.1111/j.1469-8137.2007.02041.x 17388887

[B86] Van der Putten W. H. Bardgett R. D. Bever J. D. Bezemer T. M. Casper B. B. Fukami T. (2013). Plant-soil feedbacks: the past, the present and future challenges. *J. Ecol.* 101 265–276. 10.1111/1365-2745.12054

[B87] Van Tichelen K. K. Colpaert J. V. Vangronsveld J. (2001). Ectomycorrhizal protection of *Pinus sylvestris* against copper toxicity. *New Phytol.* 150 203–213. 10.1046/j.1469-8137.2001.00081.x

[B88] Wardle D. A. Bardgett R. D. Klironomos J. N. Setälä H. Van Der Putten W. H. Wall D. H. (2004). Ecological linkages between aboveground and belowground biota. *Science* 304 1629–1633. 10.1126/science.1094875 15192218

[B89] Weemstra M. Mommer L. Visser E. J. W. van Ruijven J. Kuyper T. W. Mohren G. M. J. (2016). Towards a multidimensional root trait framework: a tree root review. *New Phytol.* 211 1159–1169. 10.1111/nph.14003 27174359

[B90] Wen Z. Shi L. Tang Y. Shen Z. Xia Y. Chen Y. (2017). Effects of *Pisolithus tinctorius* and *Cenococcum geophilum* inoculation on pine in copper-contaminated soil to enhance phytoremediation. *Int. J. Phytoremediation* 19 387–394. 10.1080/15226514.2016.1244155 27739883

[B91] White T. J. Bruns T. Lee S. Taylor J. W. (1990). “Amplification and direct sequencing of fungal ribosomal RNA genes for phylogenetics,” in *PCR Protocols: A Guide to Methods and Applications* eds Innis M. A. Gelfand D. H. Sninsky J. J. White T. J. (New York, NY: Academic Press). 10.1007/978-0-387-98141-3

[B92] Wickham H. (2009). *ggplot2: Elegant Graphics for Data Analysis.* New York, NY: Springer-Verlag 10.1016/S0045-6535(02)00232-1

[B93] Wong M. (2003). Ecological restoration of mine degraded soils, with emphasis on metal contaminated soils. *Chemosphere* 50 775–780. 10.1016/S0045-6535(02)00232-1 12688490

[B94] Wright I. J. Reich P. B. Westoby M. Ackerly D. D. Baruch Z. Bongers F. (2004). The worldwide leaf economics spectrum. *Nature* 428 821–827. 10.1038/nature02403 15103368

[B95] Zirbel C. R. Bassett T. Grman E. Brudvig L. A. (2017). Plant functional traits and environmental conditions shape community assembly and ecosystem functioning during restoration. *J. Appl. Ecol.* 54 1070–1079. 10.1111/1365-2664.12885

[B96] Zuur A. F. (2009). *Mixed Effects Models and Extensions in Ecology with R.* Berlin: Springer 10.1111/j.2041-210X.2009.00001.x

[B97] Zuur A. F. Ieno E. N. Elphick C. S. (2010). A protocol for data exploration to avoid common statistical problems. *Methods Ecol. Evol.* 1 3–14. 10.1111/j.2041-210X.2009.00001.x

