# Supplementary material for: Ectomycorrhizal Fungal Communities and Their Functional Traits Mediate Plant–Soil Interactions in Trace Element Contaminated Soils
Source: Front Plant Sci. 2018 Nov 20;9:1682. doi: 10.3389/fpls.2018.01682 (PMC6255936; doi:10.3389/fpls.2018.01682)
Supplement: Supplementary file 1 [file Data_Sheet_1.docx]

Supplementary Material

**Ectomycorrhizal fungal communities and their functional traits mediate plant-soil interactions in trace element contaminated soils**

Marta Gil-Martínez^*^, Álvaro López-García, María T. Domínguez, Carmen M. Navarro-Fernández, Rasmus Kjøller, Mark Tibbett, Teodoro Marañón

*** Correspondence:** Marta Gil-Martínez; marta.gil@irnas.csic.es

# Supplementary Figures and Tables

## Supplementary Figures


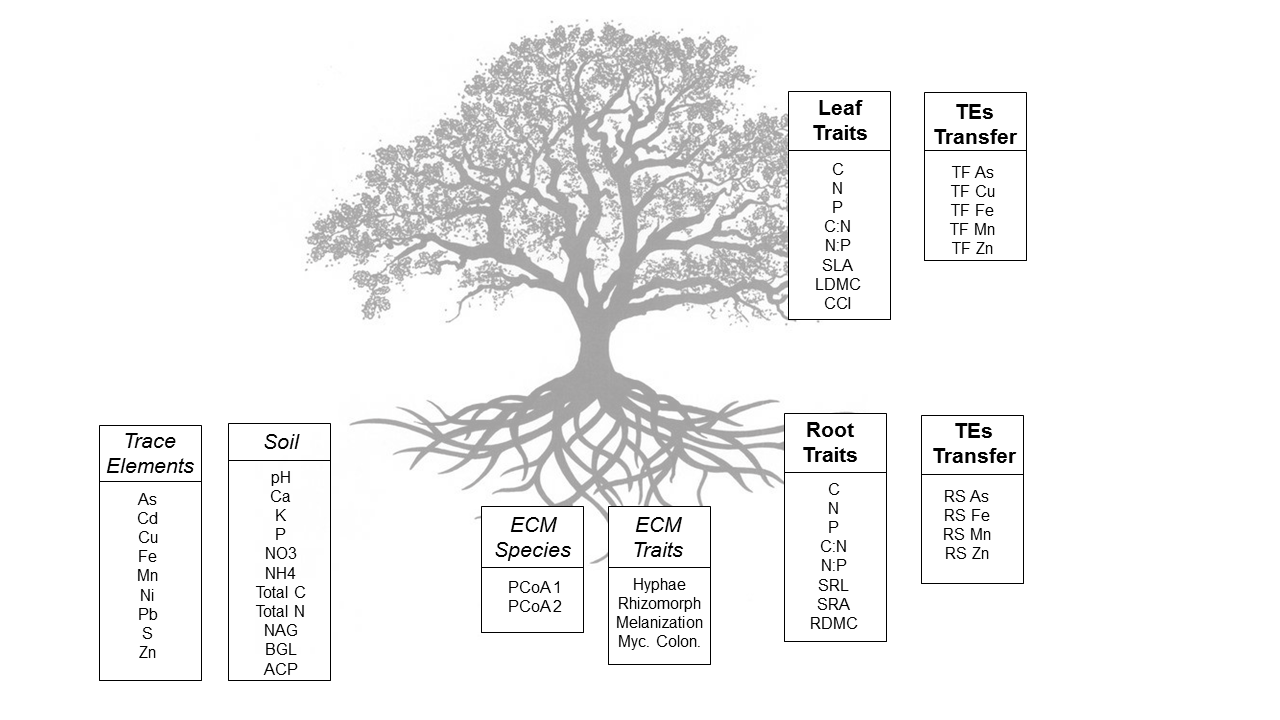


**Supplementary Figure 1.** Diagram of the predictor (trace elements, soil, ECM species and ECM traits) and response (trace elements (TEs) transfer, root traits and leaf traits) variables analysed in linear mixed models. NAG: N-acetyl-glucosaminidase; BGL: β-glucosidase; ACP: acid phosphatase; PCoA: principal coordinate analysis; Myc. Colon.: ectomycorrhizal colonization; SRL: specific root length; SRA: specific root area; RDMC: root dry matter content; RS: soil-to-root; SLA: specific leaf area; LDMC: leaf dry matter content; CCI: chlorophyll content index; TF: translocation factor.

**Supplementary Figure 2.** Species abundance distribution plot with the most abundant species taxa names.

## Supplementary Tables

**Supplementary Table 1.** Principal Coordinate Analysis (PCoA) scores per species for axis 1 and axis 2. Significant species through permutation test (*p* < 0.05*, *p* < 0.01**, *p* < 0.001***).

| **Species** | | **PCoA1** | | **PCoA2** |
| --- | --- | --- | --- | --- |
| *Astraeus_hygrometricus* | 0.093977 | | -0.18803 | |
| *Cenococcum_geophyllum* | 0.097981 | | 0.066661 | |
| *Cortinarius_belleri** | -0.13339 | | -0.25971 | |
| *Cortinarius_subbalaustinus* | -0.18172 | | -0.04428 | |
| *Cortinarius_subturibulosus* | -0.24461 | | -0.10551 | |
| *Entoloma_inusitatum* | -0.06256 | | 0.087172 | |
| *Geopora_cervina** | 0.288934 | | 0.070499 | |
| *Geopora_sp._1* | 0.053234 | | -0.0107 | |
| *Hebeloma_cavipes**** | -1.30106 | | -1.09395 | |
| *Hebeloma_cistophilum* | -0.03011 | | 0.042879 | |
| *Hymenogaster_griseus* | 0 | | 0 | |
| *Inocybe_curvipes**** | -0.16979 | | 0.305908 | |
| *Inocybe_griseovelata* | 0.196 | | 0.032404 | |
| *Inocybe_jacobi* | -0.044 | | 0.133281 | |
| *Inocybe_praetervisa* | -0.01739 | | 0.024756 | |
| *Inocybe_squamata* | -0.03771 | | -0.07163 | |
| *Laccaria_laccata* | -0.09847 | | 0.088341 | |
| *Lactarius_sp._1* | -0.00306 | | -0.06598 | |
| *Lactarius_sp._2* | -0.00306 | | -0.06598 | |
| *Melanogaster_vittadinii* | -0.02954 | | -0.01252 | |
| *Peziza_michelii* | 0.149071 | | 0.04692 | |
| *Pezizaceae_sp.* | -0.044 | | 0.133281 | |
| *Pisolithus_arhizus* | 0 | | 0 | |
| *Pisolithus_tinctorius* | -0.10681 | | 0.083641 | |
| *Pustularia_sp.* | 0.245988 | | -0.16262 | |
| *Pyronemataceae_sp._1* | 0 | | 0 | |
| *Pyronemataceae_sp._2* | 0.11051 | | 0.002073 | |
| *Pyronemataceae_sp._3* | 0.022166 | | -0.01164 | |
| *Russula_amoenolens* | 0.089316 | | -0.24717 | |
| *Russula_ilicis* | -0.1255 | | 0.008641 | |
| *Russula_insignis** | 0.324606 | | 0.067866 | |
| *Russula_praetervisa** | 0.259963 | | -0.54192 | |
| *Russula_sp.* | -0.02916 | | -0.079 | |
| *Scleroderma_cepa* | -0.13574 | | 0.064222 | |
| *Scleroderma_meridionale* | -0.04719 | | -0.0805 | |
| *Scleroderma_sp._1* | -0.05635 | | 0.041911 | |
| *Scleroderma_verrucosum* | -0.05626 | | 0.095941 | |
| *Thelephora_terrestris**** | -1.07671 | | 1.225205 | |
| *Tomentella_castanea* | 0.212718 | | 0.074104 | |
| *Tomentella_ferruginea* | 0.162295 | | 0.056792 | |
| *Tomentella_lilacinogrisea* | -0.06531 | | -0.12407 | |
| *Tomentella_sp._1* | 0.063847 | | 0.019277 | |
| *Tomentella_sp._10* | 0.061665 | | 0.020257 | |
| *Tomentella_sp._2* | -0.10581 | | 0.010034 | |
| *Tomentella_sp._3* | -0.01739 | | 0.024756 | |
| *Tomentella_sp._4* | 0.123329 | | 0.040513 | |
| *Tomentella_sp._5* | 0.174732 | | 0.003278 | |
| *Tomentella_sp._6** | 0.405988 | | 0.111256 | |
| *Tomentella_sp._8* | 0.222298 | | -0.08937 | |
| *Tomentella_sp._9* | -0.04021 | | -0.06164 | |
| *Trichophaeae_sp.* | 0.268088 | | 0.055364 | |
| *Tuber_oligospermum* | -0.03771 | | -0.07163 | |
| *Tuber_sp._1* | 0 | | 0 | |
| *Tuber_sp._2* | 0.14473 | | 0.008194 | |
| *Tuberaceae_sp._1* | 0.136643 | | -0.02444 | |

**Supplementary Table 2.** Mean and SE of soil (0-20 cm depth) chemistry and total trace elements (from López-García et al. 2018) and soil enzyme activities and available trace elements. Root and leaf nutrient concentrations, trace element concentrations and morphological traits. One-way ANOVA statistic F and *p* value. Different letters (a, b, c) represent a significant difference between sites. Lower Guideline Values (LGV) for contaminated soils are according to Ministry of the Environment Finland (2007). *Non-parametric Kruskal-Wallis H statistic with Dunn´s (Bonferroni adjusted) test.

|  | |  | | **Contamination Gradient Sites** | |  | | **ANOVA** | |
| --- | --- | --- | --- | --- | --- | --- | --- | --- | --- |
| **Nutrition** | |  | | **Site 1** | **Site 2** | **Site 3** | **Site 4** | **F** | ***p*** |
| pH | | Soil | | 4.84±0.23**c** | 6.97±0.15**a** | 6.26±0.13**b** | 7.33±0.03**a** | 51.48 | **<0.001** |
| Ca (g kg^-1^) | | Soil | | 1.89±0.27**b** | 4.89±0.09**a** | 2.19±0.52**b** | 3.24±0.41**b** | 13.39 | **<0.001** |
| (g kg^-1^) | | Root | | 10.7±1.9**c** | 25.2±2.6**a** | 10.9±1.4**bc** | 17.9±1.3**b** | 13.56 | **<0.001** |
| (g kg^-1^) | | Leaf | | 4.87±0.51 | 7.84±1.13 | 6.32±0.73 | 7.21±0.51 | 2.83 | 0.052 |
| K (mg kg^-1^) | | Soil | | 139.2±18.3**b** | 212.0±12.7**ab** | 286.1±27.9**a** | 235.9±18.4**a** | 9.25 | **<0.001** |
| (g kg^-1^) | | Root | | 3.23±0.26 | 4.09±0.42 | 3.29±0.34 | 3.56±0.31 | 1.34 | 0.278 |
| (g kg^-1^) | | Leaf | | 5.15±0.16 | 5.74±0.30 | 5.09±0.22 | 5.79±0.25 | 2.44 | 0.080 |
| P (mg kg^-1^) | | Soil | | 12.72±1.28 | 8.12±0.88 | 10.38±1.71 | 17.17±4.75 | 0.72 | 0.547 |
| (g kg^-1^) | | Root | | 0.63±0.07 | 1.08±0.18 | 0.70±0.06 | 0.87±0.10 | 6.40 | 0.094 |
| (g kg^-1^) | | Leaf | | 0.96±0.03 | 0.99±0.05 | 1.00±0.07 | 1.12±0.07 | 1.59 | 0.210 |
| NH_4_ (mg kg^-1^) | | Soil | | 4.77±0.34**a** | 2.87±0.17**b** | 5.07±0.57**a** | 3.49±0.28**b** | 10.55 | **<0.001** |
| NO_3_ (mg kg^-1^) | | Soil | | 4.78±1.35**a** | 2.49±0.45**a** | 2.64±0.47**a** | 1.21±0.19**b** | 4.27 | **0.011** |
| OC (%) | | Soil | | 1.66±0.17**a** | 1.07±0.06**bc** | 1.42±0.13**ab** | 0.85±0.07**c** | 9.38 | **<0.001** |
| (%) | | Root | | 43.09±0.92**ab** | 39.82±0.97**b** | 43.62±1.07**a** | 42.42±0.43**ab** | 3.65 | **0.021** |
| (%) | | Leaf | | 52.12±1.30**a** | 52.58±1.13**a** | 47.48±0.20**b** | 47.20±0.63**b** | 9.88 | **<0.001** |
| N (%) | | Soil | | 0.16±0.02**a** | 0.11±0.00**b** | 0.15±0.01**a** | 0.10±0.01**b** | 10.65 | **<0.001** |
| (%) | | Root | | 0.39±0.03 | 0.37±0.06 | 0.35±0.04 | 0.24±0.02 | 2.32 | 0.091 |
| (%) | | Leaf | | 1.34±0.03 | 1.29±0.04 | 1.33±0.05 | 1.33±0.07 | 0.23 | 0.877 |
| C:N | | Soil | | 10.48±0.24**a** | 9.93±0.20**a** | 9.27±0.41**ab** | 8.18±0.43**b** | 8.63 | **<0.001** |
|  | | Root | | 118.7±11.6**b** | 131.5±16.0**ab** | 141.6±14.8**ab** | 196.0±22.0**a** | 3.38 | **0.028** |
|  | | Leaf | | 38.97±1.33 | 41.31±1.86 | 36.07±1.31 | 36.36±2.32 | 1.96 | 0.137 |
| N:P | | Root | | 6.37±0.55**a** | 3.67±0.43**bc** | 5.08±0.63**ab** | 3.06±0.41**c** | 8.34 | **<0.001** |
|  | | Leaf | | 14.09±0.51 | 13.14±0.51 | 13.84±1.00 | 12.18±0.72 | 1.44 | 0.247 |
| **Enzyme Activities** (µmol PNF g^-1^ h^-1^) | | | | | |  |  |  |  |
| NAG | | Soil | | 0.332 ±0.045**b** | 0.193±0.014**c** | 0.623±0.055**a** | 0.455±0.047**ab** | 18.52 | **<0.001** |
| ΒGL | | Soil | | 0.94±0.15**b** | 1.13±0.11**b** | 1.85±0.18**a** | 0.94±0.09**b** | 9.64 | **<0.001** |
| ACP | | Soil | | 1.47±0.15**b** | 0.64±0.03**c** | 2.26±0.24**a** | 0.97±0.21**bc** | 16.29 | **<0.001** |
| **Total Trace Elements** (mg kg^-1^) | | | | |  |  |  |  |  |
|  | **LGV** | |  | |  |  |  |  |  |
| As 50 | | Soil | | 161.83±21.71**a** | 40.39±4.98**b** | 18.03±1.27**c** | 13.52±1.09**c** | 97.26 | **<0.001** |
|  | | Root | | 18.62±5.06**a** | 5.23±0.74**ab** | 3.48±0.70**b** | 2.68±0.10**b** | 27.65* | **<0.001** |
|  | | Leaf | | 0.41±0.10**b** | 0.78±0.10**ab** | 1.20±0.14**a** | 1.13±0.21**a** | 6.41 | **0.001** |
| Cd 10 | | Soil | | 0.68±0.11**a** | 0.67±0.07**a** | 0.21±0.03**b** | 0.02±0.01**c** | 43.62 | **<0.001** |
|  | | Root | | 1.78±0.15**a** | 1.20±0.18**b** | 0.82±0.11**b** | 0.40±0.03**c** | 26.57 | **<0.001** |
|  | | Leaf | | 0.18±0.05 | 0.08±0.01 | 0.08±0.02 | 0.09±0.01 | 2.36 | 0.088 |
| Cu 150 | | Soil | | 192.55±7.82**a** | 58.15±5.70**b** | 40.54±4.46**b** | 18.69±1.72**c** | 211 | **<0.001** |
|  | | Root | | 86.08±8.13**a** | 35.09±6.51**b** | 29.86±8.75**b** | 12.25±1.47**c** | 28.78 | **<0.001** |
|  | | Leaf | | 5.65±0.35**bc** | 4.75±0.15**c** | 6.98±0.30**a** | 6.09±0.33**ab** | 10.16 | **<0.001** |
| Fe | | Soil | | 40475±2136**a** | 21968±565**c** | 27522±935**ab** | 22796±1498**bc** | 27.57* | **<0.001** |
|  | | Root | | 4769±707**a** | 3238±399**b** | 3035±159**b** | 1993±133**c** | 12.93 | **<0.001** |
|  | | Leaf | | 64.38±6.63 | 54.05±6.65 | 80.25±14.73 | 63.64±11.47 | 4.36* | 0.225 |
| Mn | | Soil | | 391.5±39.5**b** | 414.8±15.4**b** | 851.9±29.6**a** | 486.4±47.8**b** | 37.09 | **<0.001** |
|  | | Root | | 135.2±15.9**ab** | 92.4±17.7**b** | 198.2±27.9**a** | 116.4±15.9**ab** | 5.26 | **0.004** |
|  | | Leaf | | 458±111**b** | 36.84±6.31**c** | 1298±219**a** | 451±85**b** | 16.55 | **<0.001** |
| Ni 100 | | Soil | | 13.01±0.70**b** | 14.60±0.44**b** | 21.69±1.04**a** | 15.73±1.23**b** | 17.44 | **<0.001** |
|  | | Root | | 5.51±0.43**ab** | 4.48±0.51**b** | 7.23±0.75**a** | 4.48±0.43**b** | 5.62 | **0.003** |
|  | | Leaf | | 1.12±0.17**a** | 0.14±0.04**b** | 1.87±0.36**a** | 0.88±0.20**a** | 10.12 | **<0.001** |
| Pb 200 | | Soil | | 274.40±37.54**a** | 76.66±8.26**b** | 57.57±6.87**b** | 19.82±1.14**c** | 89.89 | **<0.001** |
|  | | Root | | 36.63±8.96**a** | 12.09±3.06**b** | 18.01±4.19**b** | 3.32±0.68**c** | 27.48 | **<0.001** |
|  | | Leaf | | 0.36±0.04**b** | 0.30±0.07**b** | 0.82±0.17**a** | 0.36±0.04**b** | 6.40 | **0.001** |
| S | | Soil | | 3117±409**a** | 706±94**b** | 166±18**c** | 98.5±7.6**c** | 123.3 | **<0.001** |
|  | | Root | | 0.085±0.010**a** | 0.079±0.010**a** | 0.061±0.012**ab** | 0.044±0.002**b** | 21.00 | **<0.001** |
|  | | Leaf | | 0.096±0.003 | 0.090±0.003 | 0.087±0.003 | 0.090±0.003 | 1.67 | 0.191 |
| Zn 250 | | Soil | | 228.99±29.61**a** | 229.65±21.54**a** | 96.93±9.25**b** | 44.43±3.71**c** | 66.39 | **<0.001** |
|  | | Root | | 129.6±13.7**a** | 86.41±17.94**ab** | 56.47±12.34**b** | 15.26±1.06**c** | 14.07 | **<0.001** |
|  | | Leaf | | 48.63±6.33**a** | 20.61±1.63**b** | 23.19±1.60**ab** | 20.11±1.10**b** | 16.19* | **0.001** |
| **Available Trace Elements** (mg kg^-1^) | | | | |  |  |  |  |  |
| Cd | | Soil | | 0.113±0.029**a** | 0.003±0.001**b** | 0.025±0.007**ab** | 0.003±0.001**b** | 26.01* | **<0.001** |
| Cu | | Soil | | 1.70±0.60**a** | 0.082±0.004**b** | 0.072±0.011**b** | 0.044±0.005**b** | 29.04* | **<0.001** |
| Fe | | Soil | | 2.56±0.30**a** | 1.09±0.06**b** | 1.37±0.25**b** | 1.12±0.14**b** | 10.84 | **<0.001** |
| Mn | | Soil | | 19.33±5.30**a** | 0.08±0.01**c** | 22.32±6.00**a** | 1.44±0.79**b** | 29.41 | **<0.001** |
| Ni | | Soil | | 0.372±0.082**a** | 0.049±0.007**b** | 0.182±0.042**a** | 0.062±0.005**b** | 17.66 | **<0.001** |
| S | | Soil | | 329.7±141.1**a** | 11.20±1.74**b** | 9.25±2.48**bc** | 4.73±0.66**c** | 19.02 | **<0.001** |
| Zn | | Soil | | 14.35±3.58**a** | 0.28±0.16**c** | 2.52±0.95**b** | 0.12±0.02**c** | 35.21 | **<0.001** |
| **Morphological Traits** | | | | |  |  |  |  |  |
| SRA (m^2^ kg^-1^) | | Root | | 10.09±0.65 | 9.77±0.72 | 9.02±0.48 | 9.13±0.34 | 0.83 | 0.489 |
| SRL (m g^-1^) | | Root | | 5.14±0.36 | 5.31±0.43 | 4.62±0.35 | 5.42±0.30 | 0.95 | 0.426 |
| SLA (m^2^ kg^-1^) | | Leaf | | 4.83±0.16**ab** | 4.48±0.15**b** | 4.91±0.10**ab** | 5.03±0.13**a** | 2.98 | **0.044** |
| RDMC(mg g^-1^) | | Root | | 430.5±20.6 | 410.3±17.4 | 440.0±11.8 | 445.9±7.9 | 1.05 | 0.382 |
| LDMC(mg g^-1^) | | Leaf | | 53.03±0.48 | 53.94±0.54 | 53.82±0.74 | 52.29±0.43 | 1.89 | 0.149 |
| CCI (SPAD unit) | | Leaf | | 47.59±1.07 | 50.46±0.87 | 47.31±1.25 | 46.63±2.03 | 1.51 | 0.229 |
